# UnionCAM: enhancing CNN interpretability through denoising, weighted fusion, and selective high-quality class activation mapping

**DOI:** 10.3389/fnbot.2024.1490198

**Published:** 2024-11-14

**Authors:** Hao Hu, Rui Wang, Hao Lin, Huai Yu

**Affiliations:** ^1^The Institute of Computing, China Academy of Railway Sciences Corporation Ltd, Beijing, China; ^2^The Center of National Railway Intelligent Transportation System Engineering and Technology, Beijing, China; ^3^Xi'an Jiaotong University, Xi'an, China; ^4^Signal and Communication Research Institute, China Academy of Railway Sciences Corporation Ltd, Beijing, China

**Keywords:** visual interpretation, class activation map, CNN, Union Class Activation Mapping, denoised CAMs, region-based CAMs

## Abstract

Deep convolutional neural networks (CNNs) have achieved remarkable success in various computer vision tasks. However, the lack of interpretability in these models has raised concerns and hindered their widespread adoption in critical domains. Generating activation maps that highlight the regions contributing to the CNN's decision has emerged as a popular approach to visualize and interpret these models. Nevertheless, existing methods often produce activation maps contaminated with irrelevant background noise or incomplete object activation, limiting their effectiveness in providing meaningful explanations. To address this challenge, we propose Union Class Activation Mapping (UnionCAM), an innovative visual interpretation framework that generates high-quality class activation maps (CAMs) through a novel three-step approach. UnionCAM introduces a weighted fusion strategy that adaptively combines multiple CAMs to create more informative and comprehensive activation maps. First, the denoising module removes background noise from CAMs by using adaptive thresholding. Subsequently, the union module fuses the denoised CAMs with region-based CAMs using a weighted combination scheme to obtain more comprehensive and informative maps, which we refer to as fused CAMs. Lastly, the activation map selection module automatically selects the optimal CAM that offers the best interpretation from the pool of fused CAMs. Extensive experiments on ILSVRC2012 and VOC2007 datasets demonstrate UnionCAM's superior performance over state-of-the-art methods. It effectively suppresses background noise, captures complete object regions, and provides intuitive visual explanations. UnionCAM achieves significant improvements in insertion and deletion scores, outperforming the best baseline. UnionCAM makes notable contributions by introducing a novel denoising strategy, adaptive fusion of CAMs, and an automatic selection mechanism. It bridges the gap between CNN performance and interpretability, providing a valuable tool for understanding and trusting CNN-based systems. UnionCAM has the potential to foster responsible deployment of CNNs in real-world applications.

## 1 Introduction

Deep learning models have revolutionized various domains, such as computer vision, natural language processing, and speech recognition. However, as these models become increasingly complex and opaque, the interpretation of their decision-making processes has become crucial for building trust and ensuring reliability. Among the various interpretation methods, visualizing feature maps or learned weights is the most intuitive and convincing approach for users to understand the reasoning behind the model's predictions. In convolutional neural networks (CNNs), which have become the primary choice for feature extraction in computer vision, gradient-based interpretation (Simonyan and Zisserman, 2014), region-based visualization (Wang et al., 2020b), and Class Activation Mapping (CAM) (Zhou et al., 2016) are the most widely used methods for explaining convolutional operations.

Gradient-based approaches, such as Simonyan and Zisserman (2014), Adebayo et al. (2018), Omeiza et al. (2019), Springenberg et al. (2014), Sundararajan et al. (2017), and Zeiler and Fergus (2014), backpropagate the gradient of the target class to the input layer, highlighting image regions that significantly impact the prediction. However, these methods often generate noisy and incomplete activation maps, focusing primarily on edge or texture features while neglecting fine-grained information. Moreover, the gradients of CNNs may vanish or explode due to the saturation problem in the activation functions, such as Sigmoid or ReLU (Zhang et al., 2021b), further compromising the quality of the activation maps.

CAM (Zhou et al., 2016) and its extensions, such as GradCAM (Selvaraju et al., 2017) and GradCAM++ (Chattopadhay et al., 2018), provide visual explanations by linearly combining weighted activation maps from convolutional layers. Despite their effectiveness, these methods have limitations: CAM is architecture-sensitive and requires modifying the network structure, while GradCAM and GradCAM++ may activate irrelevant parts, such as the background, due to gradient noise. Furthermore, these methods may generate incomplete activation maps that fail to capture the entire object of interest, as they rely on the gradients of the target class, which may not cover all the discriminative regions.

Region-based methods, such as ScoreCAM (Wang et al., 2020b) and GroupCAM (Zhang et al., 2021a), calculate the importance of activation maps using the category confidence of corresponding input features rather than local region gradients. Although these methods can effectively remove background areas, they may generate incomplete activation maps and have high computational costs. Moreover, these methods do not fully exploit the information from the gradients, which can provide valuable insights into the model's decision-making process.

To address these limitations and provide a more accurate and comprehensive visual interpretation of deep CNNs, we propose UnionCAM, a novel method that employs a “denoising-union-selection” strategy to generate class activation maps. The main contributions of this paper are as follows:

- To effectively remove background noise from gradient-based activation maps and mitigate challenges such as gradient noise and vanishing gradients, we introduce the Activation Map Denoising (AMD) module. It applies a denoising function to the gradients, which enables the AMD module to better capture discriminative regions by generating more accurate and reliable activation maps.
- We propose the Activation Map Union (AMU) module, combining the denoised activation maps from AMD with region-based activation maps, to integrate the advantages of gradient-based and region-based methods. AMU generates more complete and informative activation maps by capturing both fine-grained details and global context, offering a more comprehensive understanding of the model's decision-making process.
- To select the most informative activation map from the union set generated by AMU, We further develop the Activation Map Selection (AMS) module. AMS employs a novel scoring function that considers both the discriminative power and the spatial consistency of the activation maps, ensuring that the selected map provides the most accurate and reliable visual interpretation. This module further enhances the interpretability and trustworthiness of the generated explanations.
- Through extensive experiments on various benchmarks, we demonstrate that UnionCAM achieves state-of-the-art performance in visual interpretation, outperforming existing methods in terms of both accuracy and completeness. UnionCAM effectively addresses the problems of incomplete activation and background activation, providing a more trustworthy and interpretable visualization of deep CNNs. The superior performance of UnionCAM highlights its potential for facilitating the understanding and debugging of deep learning models in real-world applications.

## 2 Related work

Feature or weight visualization enhances model transparency and understanding by illustrating how decisions are made. It aids in understanding the human brain, facilitates early diagnosis of conditions, improves the accuracy of prediction systems, and helps detect potential failures, among other benefits (Zong et al., 2024; Yu et al., 2022). CAM (Zhou et al., 2016) is one of the pioneering works that uses a weighted sum of the feature maps from the last convolutional layer to generate class-specific activation maps, which has inspired numerous subsequent developments in the field. In this paper, we reviewed recent relevant works and categorized them into three types: gradient-based, gradient-free, and ensemble methods. Additionally, some feature visualization methods, such as GAN-based approaches, can also provide valuable methods for understanding and interpreting model behavior.

### 2.1 Gradient-based methods

Gradient-based methods utilize the gradients of the model's output with respect to the input or intermediate feature maps to highlight the important regions. Grad-CAM (Selvaraju et al., 2017) generalizes CAM to models without global average pooling by using the gradients of the target class score with respect to the feature maps. Expanding on this work, a range of gradient-based methods have been developed to enhance granularity using various approaches, such as GradCAM++ (Chattopadhay et al., 2018), Smooth GradCAM++ (Omeiza et al., 2019), XGradCAM (Fu et al., 2020), Augmented GradCAM (Morbidelli et al., 2020), Integrated GradCAM (Sattarzadeh et al., 2021), and among others. LayerCAM (Jiang et al., 2021) enhances the reliability of CAMs by incorporating information from various layers through weighted aggregation, offering a more detailed coarse-to-fine aggregation solution. Despite their computational efficiency, gradient-based methods may capture irrelevant information in the activation maps since the feature maps are not always related to the target class (Zhang et al., 2021b).

### 2.2 Gradient-free methods

Gradient-free CAMs, on the other hand, aim to identify the importance of different input regions by occluding or perturbing them and observing the effect on the model's output (Zhang et al., 2021b; Selvaraju et al., 2017; Kapishnikov et al., 2019; Zhang et al., 2018; Liu et al., 2021b; Yan et al., 2021; Ahn et al., 2019; Liu et al., 2021a; Liang et al., 2022; Li et al., 2021; Cui et al., 2021; Ranjan et al., 2019; Lu et al., 2023; Jiao et al., 2018). One of the earliest works, RISE (Petsiuk et al., 2018), generates random binary masks to occlude different parts of the input image for prediction scores, and then uses a linear combination of these masks and corresponding scores to obtain the final importance map. Although effective, it is inefficient due to the need for thousands of random masks. ScoreCAM (Wang et al., 2020b) improves upon RISE by using the activation maps as the initial masks and combining them with the model's output scores to generate more accurate activation maps, spearheading the advancement of methods such as Smooth ScoreCAM (Wang et al., 2020a), Integrated ScoreCAM (Naidu et al., 2020), FIMF ScoreCAM (Li et al., 2023), GroupCAM (Zhang et al., 2021a), and etc. Differently, AblationCAM (Ramaswamy et al., 2020) utilizes the effective slope which is characterized as the difference between the original prediction score and the prediction score derived from an ablated activation map; based on this work, AblationCAM++ (Salama et al., 2022) further introduce clustering to group activation maps for improved efficiency. ReciproCAM (Byun and Lee, 2022) significantly accelerates execution speed by using the reciprocal relationship between activation maps and predictions, further inspiring the development of ViT-ReciproCAM (Byun and Lee, 2023) for Vision Transformers (ViT). Although Gradient-Free CAMs generally produce more human-interpretable explanations, they may generate incomplete activation maps due to the presence of salient regions that are not necessarily related to the target class.

### 2.3 Ensemble methods

To address the limitations of gradient-based and gradient-free methods, certain approaches FDCAM (Li et al., 2022) combine gradient-based and score-based weights to derive CAM's weightings, harnessing the strengths of both techniques. Feature CAM (Clement et al., 2024) combines perturbation and activation solutions for fine-grained, class-discriminative visualizations. Grad++-ScoreCAM (Soomro et al., 2024) enhances CNN interpretability and localization by first generating a coarse heatmap with GradCAM++ and then refining it with ScoreCAM to incorporate intermediate layer information. Our proposed method UnionCAM also falls in this part, by denoising the gradient-based activation maps and then merging them with the region-based maps using a learned weight, UnionCAM generates more accurate and complete visual explanations. In the following sections, we will describe the proposed method in detail and demonstrate its effectiveness through comprehensive experiments.

### 2.4 Feature visualization via generation methods

Methods based on generative models also play an important role in feature visualization. GAN functions as an insightful method that clarifies the decision-making process and offers effective support for diverse tasks (Bau et al., 2018; Yu et al., 2022; Lang et al., 2021). Bau et al. (2018) introduce an analytical framework for visualizing and understanding GANs at the levels of units, objects, and scenes. Lang et al. (2021) train a generative model to clarify the various attributes that contribute to classifier decisions. Yu et al. (2022) propose the multidirectional perception generative adversarial network (MP-GAN) to visualize morphological features for whole-brain MR images. Besides, diffusion model-based feature visualization methods provide visualization strategies from a different perspective. VPD (Zhao et al., 2023) proposes to refine text features and prompt the denoising decoder for better interaction between visuals and text, using cross-attention maps for guidance. NeuroDM (Qian et al., 2024) first extracts the visual-related features with high classification accuracy from EEG signals by EV-Transformer, and then employs EG-DM to synthesize high-quality images with the EEG visual-related features.

## 3 Methodology

**Figure 1:**
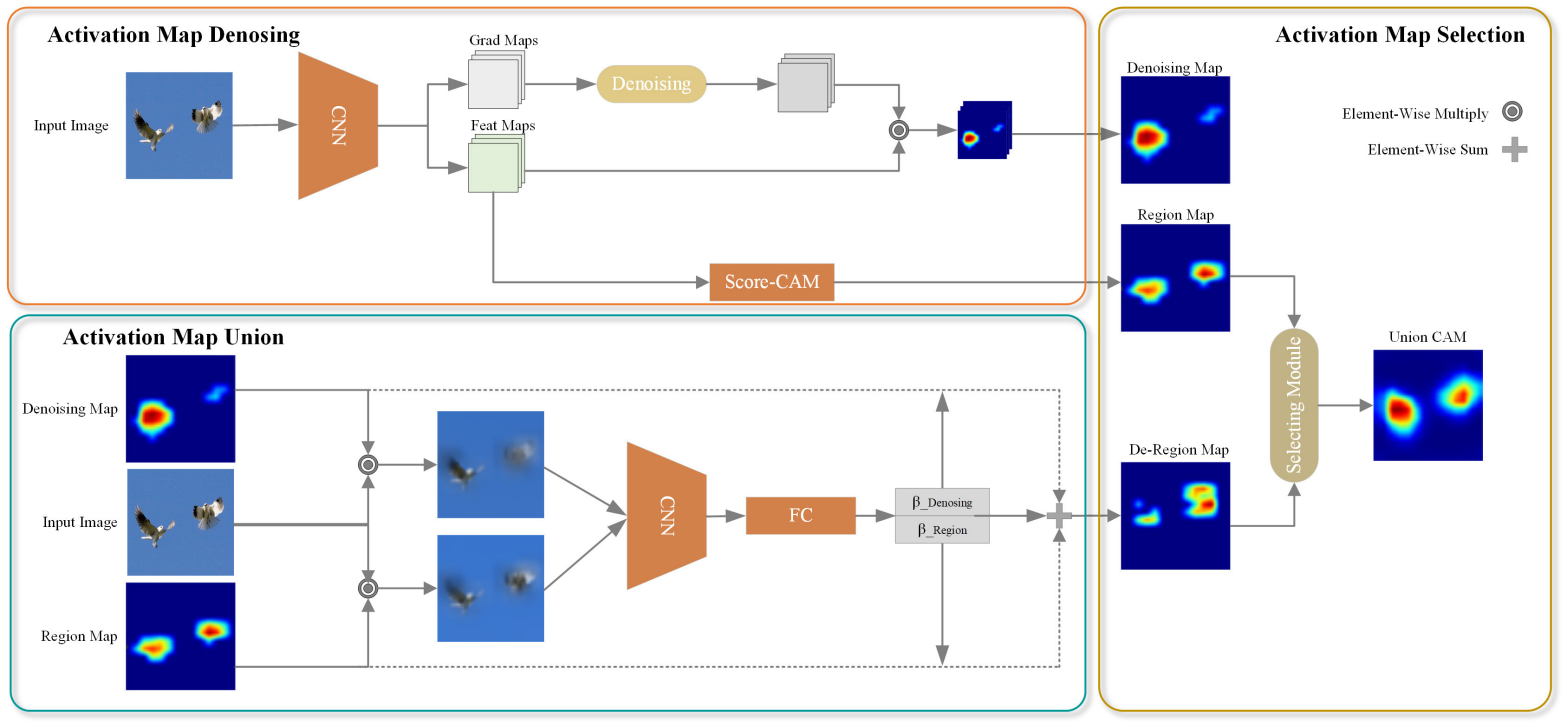
Pipeline of UnionCAM. AMD module is used to denoise meaningless background to generate a purer CAMs. Then, AMU module is to generate a complete CAM, which takes the class confidence as the weight to union the denoised CAMs and the region-based CAMs. AMS module is used to select a better interpretation effect of CAMs. ⊙ denotes element-wise multiple operation, which used for weight and feature maps. ⊕ represents add operation.

The overall architecture of the proposed UnionCAM is illustrated in Figure 1, and we also present the pseudocode in Algorithm 1. This section provides a detailed explanation of the three key modules in the proposed method: Activation Map Denoising (AMD), Activation Map Union (AMU), and Activation Map Selection (AMS). Let $I_{0}\in {\mathbb{R}}^{3\times M\times N}$ be an input image, where *M* and *N* represent the height and width of the image, respectively. Let $I_{b}\in {\mathbb{R}}^{3\times M\times N}$ be a black image with the same dimensions as *I*_0_. We denote *f*(·) as a deep neural network which predicts a score $y^{c}=f^{c}\left(I_{0}\right)\in \mathbb{R}$ for class *c* given an input image *I*_0_.

**Algorithm 1:**
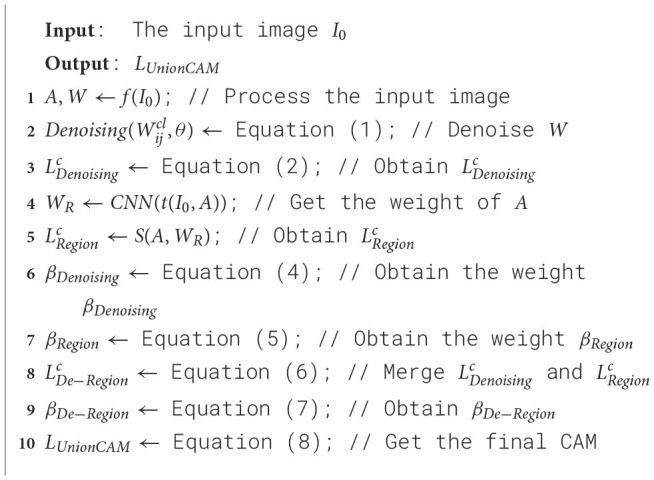
UnionCAM.

### 3.1 Activation map denoising

After the feature extraction backbone network, the feature map and the corresponding reverse gradient of each channel can be obtained, as shown in the “Feat Maps” and “Grad Maps” in Figure 1. However, the gradients of CNNs may be noisy and even tend to disappear due to the saturation problem of the zero gradient region of the “Sigmoid” or “ReLU” function (Zhang et al., 2021b). To address this issue, we propose an activation map denoising (AMD) method, as illustrated in the “Activation Map Denoising” part in Figure 1. This subsection will elaborate on this module. The AMD module mainly designs a function to denoise the gradient obtained after the backbone network. For the convenience of explanation, the gradient is denoted as *W* here.

For each channel of *W*, the θ percentile is calculated as the denoising threshold. If the gradient value is greater than or equal to the threshold, the gradient value at the corresponding position remains unchanged; otherwise, the gradient value at the corresponding position is set to 0. This denoising operation is reasonable because positions with relatively small gradient values have a high probability of being background areas unrelated to the detection target. In this way, we can remove detect target-independent background regions, thereby improving the localization effect of class activation maps on detected targets.

In addition to an illustration of the denoising process in Figure 1, we formulate the denoising function in this section. For a scalar *W*_*ij*_ in *W*, the denoising function can be formulated as:


(1) $$Denoising(W_{ij}^{cl},\theta )=\{\begin{array}{ll} W_{ij}^{cl}, & W_{ij}^{cl}\geq p(W^{cl},\theta );\\ 0, & otherwise, \end{array}$$

where *p*(*W*^*cl*^, θ) calculates the θ percentile of the *l*-th layer *W*^*cl*^ for specific category *c*. With denoised weighting maps, the class related feature maps are defined as the weighted sum to obtain the class activation map, which can be formulated as,


(2) $$\begin{array}{l} L_{Denoising}^{c}=\underset{l}{\sum }\alpha^{cl}○\text{ReLU}\left(W^{cl}\right)○A^{l}, \end{array}$$

where ° is Hadamard product, *A*^*l*^ is the feature map of the *l*-th layer. The weight α is the pixel-level average coefficient, which is defined as:


(3) $$\alpha_{ij}^{cl}=\{\begin{array}{ll} \frac{1}{\sum_{m,n}\left(W_{mn}^{cl}\text{I}(W_{mn}^{cl})\right)} & \text{if }W_{ij}^{cl}>0;\\ 0 & \text{otherwise}. \end{array}$$

where 𝕀(·) is an indicator function checking whether the given variable is >0, and $W_{ij}^{cl}$ is the gradient value corresponding to the (*i, j*) position in the denoised gradient *W* of the *l*-th channel. The locations where the gradient values are >0 are most likely the locations of the target. The use of pixel-level average coefficients can avoid excessive channel weights in small activation areas, which will lead to significant activation problems. After the above process, the gradient-based class activation map after denoising can be obtained, which is denoted as $L_{Denoising}^{c}$. A high-quality $L_{Denoising}^{c}$ serves as the basis for the upcoming soft and hard integration strategy, ensuring that the model can effectively leverage refined features.

### 3.2 Activation map union

Gradient-based CAM introduces noise due to the gradient. Although the denoising method in Section 3.1 can remove part of the noise, it cannot completely eliminate the background area unrelated to the target class. To further suppress the background area, we draw inspiration from the area-based method. In our approach, the feature map of each channel is used as a mask to activate the corresponding area in the original image. The activated area is then used as the input to the CNN, and the prediction score is used as the weight of the feature map. The weighted summation of these feature maps yields the class activation maps, denoted as $L_{Region}^{c}$.

By using $L_{Region}^{c}$, the influence of the gradient on the class activation map is significantly reduced, and the background area can be effectively suppressed. However, for targets with distinctive features, the main part of the target may also be partially removed, leading to an incomplete class activation map. To address this issue and obtain a more complete representation of the main object while further suppressing the background, we propose a method to combine $L_{Denoising}^{c}$ and $L_{Region}^{c}$. The two class activation maps are merged using weights β_*Denoising*_ and β_*Region*_ for $L_{Denoising}^{c}$ and $L_{Region}^{c}$, respectively. The overall process is illustrated in the “Activation Map Union” block of Figure 1. In the following, we formulate this module in detail.

To combine the two types of activation maps using weights, we first need to determine their respective weights. The weight β_*Denoising*_ is formulated as:


(4) $$\begin{array}{l} \beta_{Denoising}=f^{c}\left(L_{Denoising}^{c}○I_{0}\right)-f^{c}\left(I_{b}\right), \end{array}$$

Here, we perform the ° operation on the denoised CAM $L_{Denoising}^{c}$ and the original image, which means that $L_{Denoising}^{c}$ is used as the mask to activate the corresponding part of the original image.

$f^{c}\left(L_{Denoising}^{c}○I_{0}\right)$ denotes the activation image generated by using $L_{Denoising}^{c}$ as the mask and inputting it into the convolutional neural network for the corresponding target category *c*, and $f^{c}\left(I_{b}\right)$ represents the score corresponding to the target category c obtained by inputting the all-black image *I*_*b*_ into the convolutional neural network. Therefore, β_*Denoising*_ can be understood as the contribution of the $L_{Denoising}^{c}$ activation area to the score of the target category *c*. Similarly, β_*Region*_ can be understood as the contribution of the $L_{Region}^{c}$ activation region to the target category *c*, which can be formulated as:


(5) $$\begin{array}{l} \beta_{Region}=f^{c}\left(L_{Region}^{c}○I_{0}\right)-f^{c}\left(I_{b}\right), \end{array}$$

where $f^{c}\left(L_{Region}^{c}○I_{0}\right)$ denotes the score of the target category *c* obtained by inputting the activation image generated using $L_{Region}^{c}$ as the mask into the convolutional neural network, and $f^{c}\left(I_{b}\right)$ represents the score of the target category *c* obtained by inputting the all-black image *I*_*b*_ into the convolutional neural network.

Having obtained the score contributions β_*Denoising*_ and β_*Region*_ of the $L_{Denoising}^{c}$ and $L_{Region}^{c}$ activation regions to the target category *c*, respectively, we can merge the two types of activation maps using these contributions as weights:


(6) $$\begin{array}{l} L_{De-Region}^{c}=\beta_{Denoising}\cdot L_{Denoising}^{c}+\beta_{Region}\cdot L_{Region}^{c}. \end{array}$$

By combining the two activation maps weighted by their respective contributions to the target category score, the resulting class activation map emphasizes the target object's main area (high-scoring part) in the original image while suppressing the background area (low-scoring part). This soft integration strategy enables the model to adaptively acquire meaningful features while enhancing its ability to understand and process complex data patterns. This approach helps to obtain a more complete representation of the target object while effectively reducing background activation, thereby improving the interpretability and localization accuracy of the class activation map.

### 3.3 Activation map select

The combination of the two activation maps using their respective scores as weights, as described in Section 3.2, does not always guarantee an improved explanatory power of the resulting activation map. One potential scenario is when the background area outside the target object in $L_{Denoising}^{c}$ is not entirely suppressed, and the weight β_*Denoising*_ obtained from the CNN is greater than β_*Region*_. In this case, merging the two activation maps with the scores as weights may introduce redundant background components, which can negatively impact the final interpretation and localization accuracy of the class activation map.

**Figure 2:**
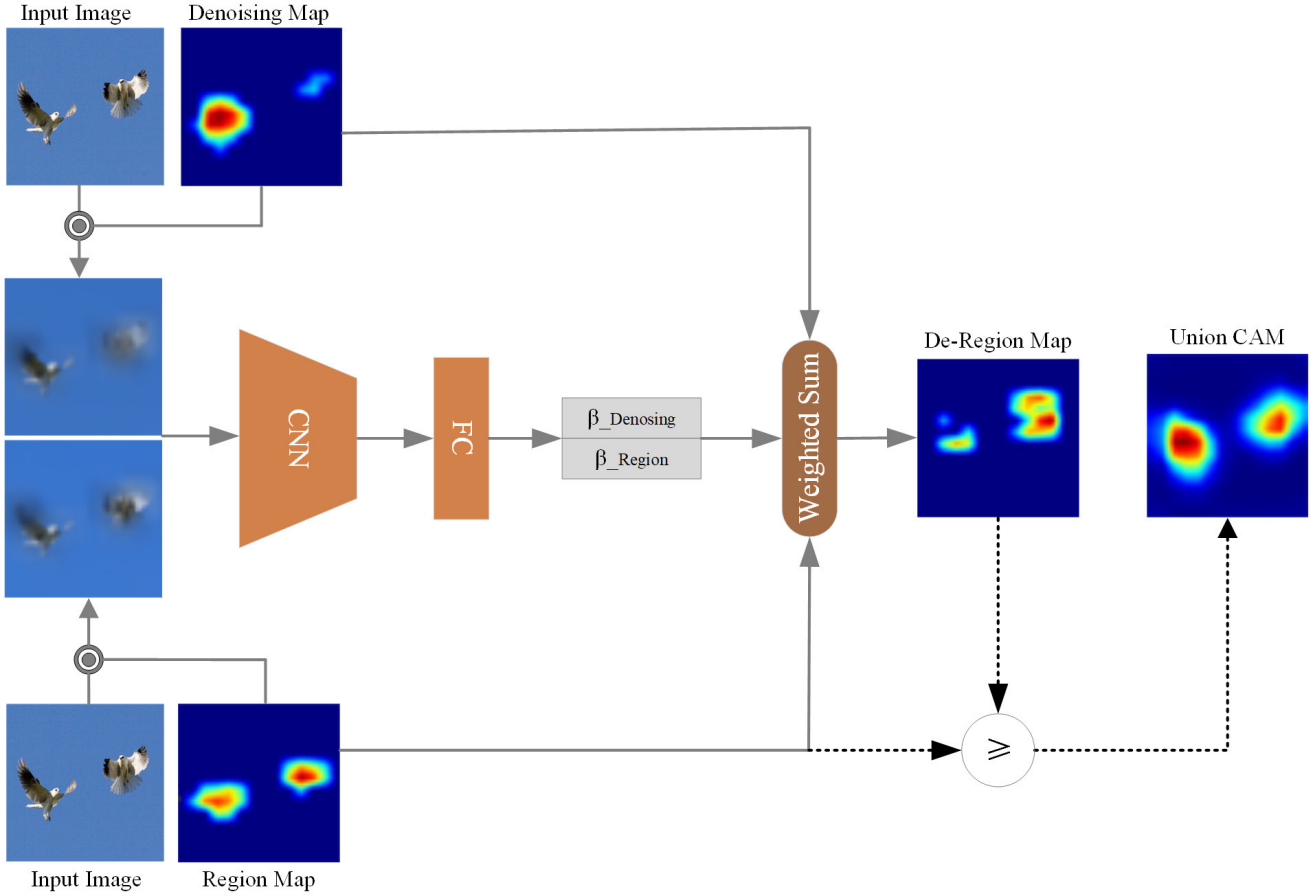
Activation map select. Based on the comparison between *β*_*De*−*Region*_ and *β*_*Region*_, we select the corresponding element from De-Region Map and Region Map to form the Union CAM.

To mitigate the above issue, we propose the Activation Map Selection (AMS) method. Considering both $L_{De-Region}^{c}$ and $L_{Region}^{c}$, AMS can choose the class activation map that provides a more interpretable representation of the target category. This capability enables AMS to select the CAM that yields a higher score for the target category, indicating better localization and interpretation of the target object. The overall workflow of the AMS method is illustrated in Figure 2.

We subsequently formulate AMS, based on the score contribution β_*Region*_ of the $L_{Region}^{c}$ activation region to the target category c has been obtained from Equation 5 and the combined class activation map $L_{De-Region}^{c}$ is also obtained from Equation 6. To select the CAMs according to the interpretability of the target category, we must first get the score contribution β_*De*−*Region*_ of the $L_{De-Region}^{c}$ activation region to the target category c. Similarly, *w*_*De*−*Region*_ can be formulated as:


(7) $$\begin{array}{l} \beta_{De-Region}=f^{c}\left(L_{De-Region}^{c}○I_{0}\right)-f^{c}\left(I_{b}\right) \end{array}$$

After obtaining the score contribution β_*De*−*Region*_ of the $L_{De-Region}^{c}$ activation region to the target category c, we can select the final CAM result according to the bigness of β_*De*−*Region*_ and β_*Region*_ and its decision-making process can be formulated as:


(8) $$L_{UnionCAM}^{c}=\{\begin{array}{ll} L_{De-Region}^{c} & \text{if }\beta_{De-Region}>\beta_{Region};\\ L_{Region}^{c} & \text{otherwise}. \end{array}$$

As a combination of soft and hard selection strategy, AMS enables a more flexible dynamic integration of both gradient-based activation maps and region-based activation maps, dynamically adapting to different input characteristics. The β_*Denosing*_ and β_*Region*_ first softly select the denoising map and region map for integration, which sometimes can introduce noise signals, thus blurring the decision-making process. Compensatorily, Equation 8 offers a hard selection to alleviate this issue, promoting the model to make more reliable decisions, which enhances this dynamic adaptability by more effectively capturing activation regions that are beneficial to the decision-making process.

## 4 Experiments

In this section, we conduct experiments to evaluate the effectiveness of the proposed interpretation method. First, we provide a basic description of the datasets and data preprocessing for the experiments in Section 4.1. Second, in Section 4.2, we quantitatively evaluate UnionCAM against other mainstream class activation map methods using established evaluation metrics. Then, we qualitatively evaluate our method with visualizations on the ILSVRC2012 (Russakovsky et al., 2015) in Section 4.3. Finally, in Section 4.4, we assess the effectiveness of each module proposed in this paper through ablation experiments.

### 4.1 Experimental setup

**Figure 3:**
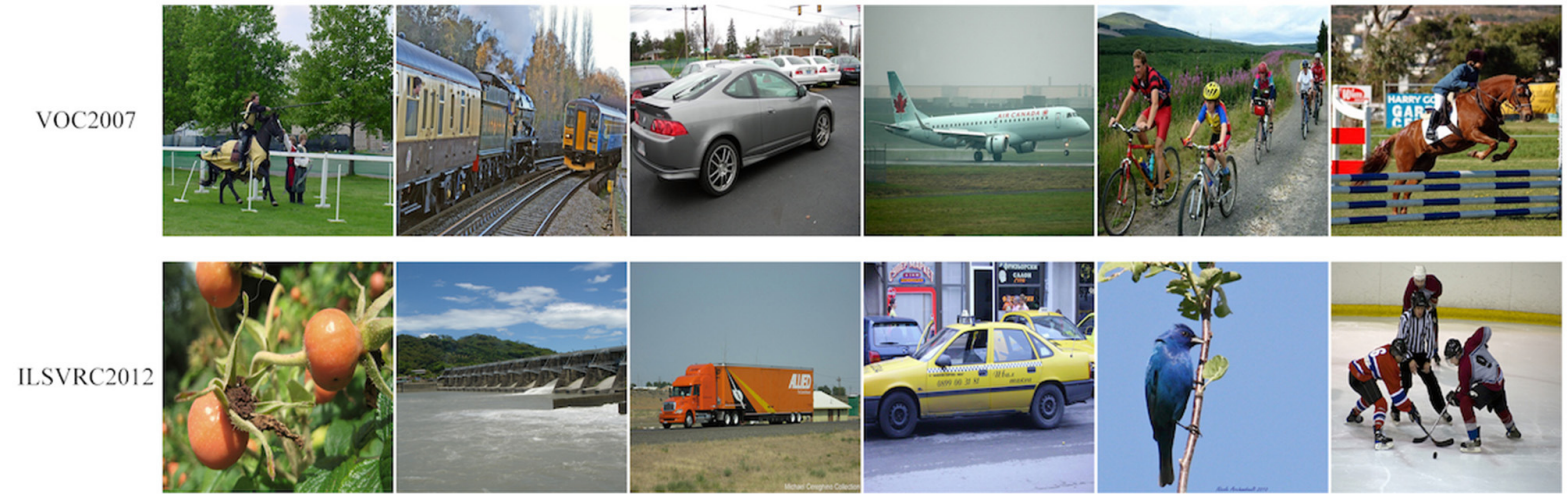
Examples from the ILSVRC2012 and VOC2007 datasets.

Experiments are performed on commonly used computer vision datasets, including the validation set of ILSVRC2012 (Russakovsky et al., 2015) and the VOC2007 test set (Everingham et al., 2015), as shown in Figure 3. For both datasets, all images were resized to 3 × 224 × 224, then converted to tensors, and normalized to the range [0,1]. No additional preprocessing was applied. We utilize the pretrained torchvision model VGG16 (Simonyan and Zisserman, 2014) as the base classifier model. Unless stated otherwise, the θ parameter in UnionCAM is set to 10. To ensure a fair comparison, all activation maps are upsampled to 224 × 224 by using bilinear interpolation.

### 4.2 Quantitative evaluation of evaluation indicators

We initially evaluate the confidence of the activation maps generated by UnionCAM for the object recognition task employed in Chattopadhay et al. (2018). The original input activates specified regions in the given image through point-wise multiplication with activation maps to observe score changes in the target class. We adopt the metric from Chattopadhay et al. (2018), where the average drop is formulated as: $\overset{N}{\underset{i=1}{\sum }}\frac{\max\left(0,\overset{c}{\underset{i}{y}}-o_{i}^{c}\right)}{y_{i}^{c}}\times 100$, and the average increase is formulated as: $\overset{N}{\underset{i=1}{\sum }}\frac{\text{Sign}\left(y_{i}^{c}<o_{i}^{c}\right)}{N}\times 100$. Here, $y_{i}^{c}$ denotes the score of category *c* predicted after inputting the original image into the network, and $o_{i}^{c}$ denotes the score predicted after the activation map activates certain parts of the original image. *Sign* is an indicator function that returns 1 if the input condition is true. Experiments are performed on the ImageNet (ILSVRC2012) validation set with 2,000 images randomly selected. Our algorithm consumes 2.22 GB of memory during operation, and the average processing time per image is 1.16 s, which is evaluated on an NVIDIA RTX A6000 GPU. The results are summarized in Table 1. Similarly, the experimental results on the VOC2007 test set are shown in Table 2.

**Table 1:** Recognition evaluation results on the ILSVRC2012 dataset (the smaller the average drop, the better, and the larger the average increase, the better).

**Method**	**GradCAM**	**GradCAM++**	**ScoreCAM**	**GroupCAM**	**UnionCAM**
Average drop (%)	72.30	67.62	56.11	63.46	**43.15**
Average increase (%)	19.45	16.35	22.7	21.4	**28.95**

The bold values indicate evaluation metric of the activation maps confidence.

**Table 2:** Recognition evaluation results on the VOC2007 dataset (the smaller the average drop, the better, and the larger the average increase, the better).

**Method**	**GradCAM**	**GradCAM++**	**ScoreCAM**	**GroupCAM**	**UnionCAM**
Average Drop(%)	53.07	39.51	18.88	32.33	**15.77**
Average Increase(%)	22.15	10.72	27.41	25.62	**28.57**

The bold values indicate evaluation metric of the activation maps confidence.

As shown in Table 1, the average drop rate and average increase rate of UnionCAM are 43.15 and 28.95%, respectively, which are superior to the previous methods. Good performance on recognition tasks shows that UnionCAM is able to successfully find the most recognizable regions of the target object, not just what humans consider important. Experimental results on recognition tasks show that UnionCAM can more realistically reveal the decision-making process of the original CNN model than previous methods.

In addition, to more fully explain the superiority of our method, we also evaluate the deletion and insertion metrics mentioned in Petsiuk et al. (2018). This metric is in addition to the Average Decline and Increase metrics. The removal metric measures the decreasing trend of the predicted category score by removing more and more important pixels from the original image using the activation map as a mask. A sharp drop will cause the area under the curve to become smaller, and the smaller the area under the curve, the better the interpretation of the activation map. The insertion metric is just the opposite, as more and more pixels are inserted into the input image, the predicted class score rises. The larger the area under the curve, the better the interpretation of the activation map.

**Figure 4:**
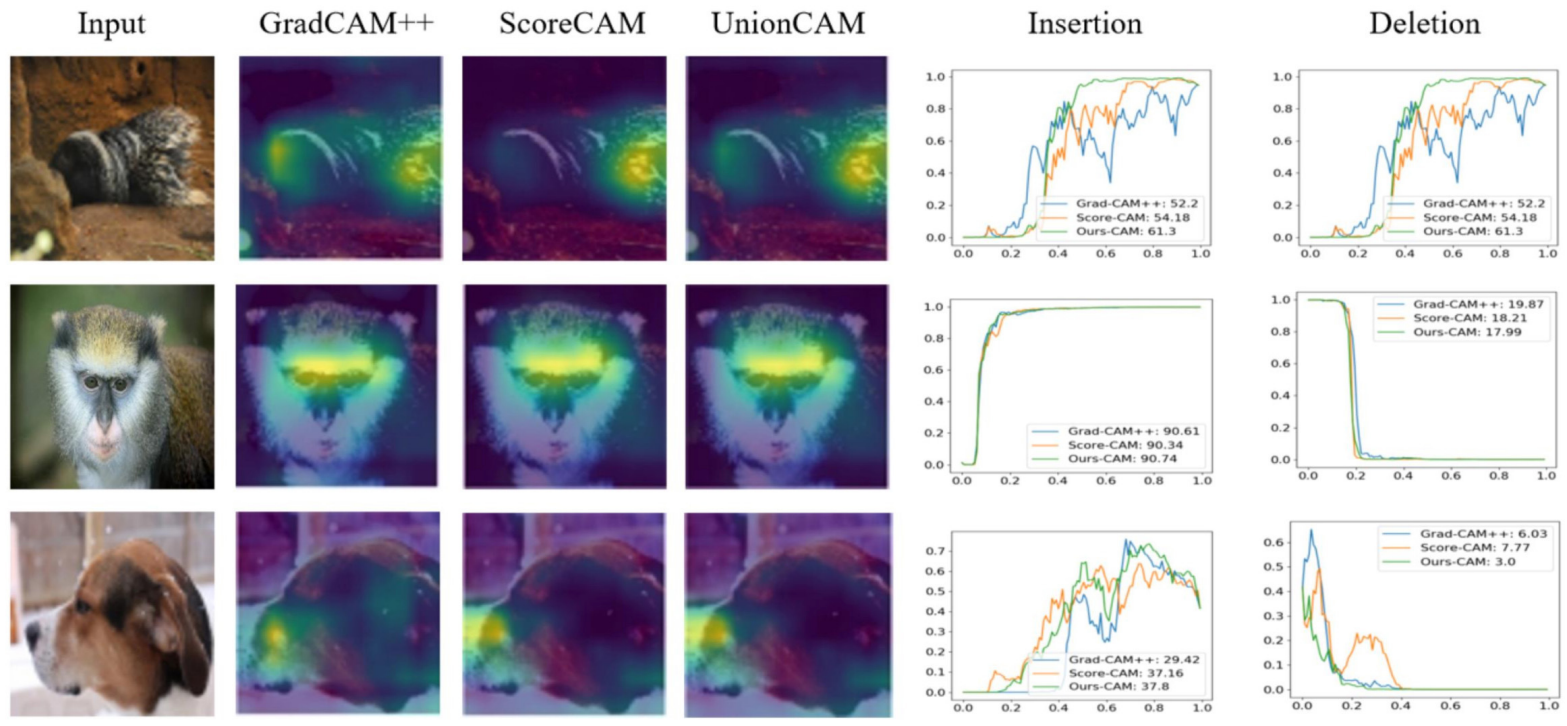
GradCAM++, ScoreCAM, and UnionCAM (ours) generate activation maps for representative images, respectively. And generate deletion and insertion curves according to the activation map, in the insertion curve, the prediction score grows faster, and in the deletion curve, the classification confidence decreases faster, which is a better explanation.

There are several methods (Dabkowski and Gal, 2017) for removing pixels from an image, all of which have different advantages and disadvantages. We took the same approach as Zhang et al. (2021a). We calculate the AUC of the classification score after Softmax as a quantitative measure. In addition, we calculated the over-all score composite evaluation deletion and insertion results, calculated as AUC(insertion)—AUC(deletion). The sample pictures are shown in Figure 4, and the average results calculated by randomly selecting 2,000 pictures on the ImageNet (ILSVRC2012) validation set are shown in Table 3. Our method achieves the best results.

**Table 3:** In the ImageNet (ILSVRC2012) validation set, comparisons are made in terms of deletion (lower is better), insertion (higher is better) scores and over-all (**higher is better**) evaluation metrics.

**Method**	**GradCAM**	**GradCAM++**	**ScoreCAM**	**GroupCAM**	**UnionCAM**
Insertion (%)	53.5	50.0	55.1	56.8	**57.2**
Deletion (%)	13.3	14.8	**11.5**	12.3	11.9
Over-all (%)	40.2	35.2	43.6	44.5	**45.3**

The best results are marked in bold. The best results are marked in bold.

### 4.3 Visual qualitative evaluation

**Figure 5:**
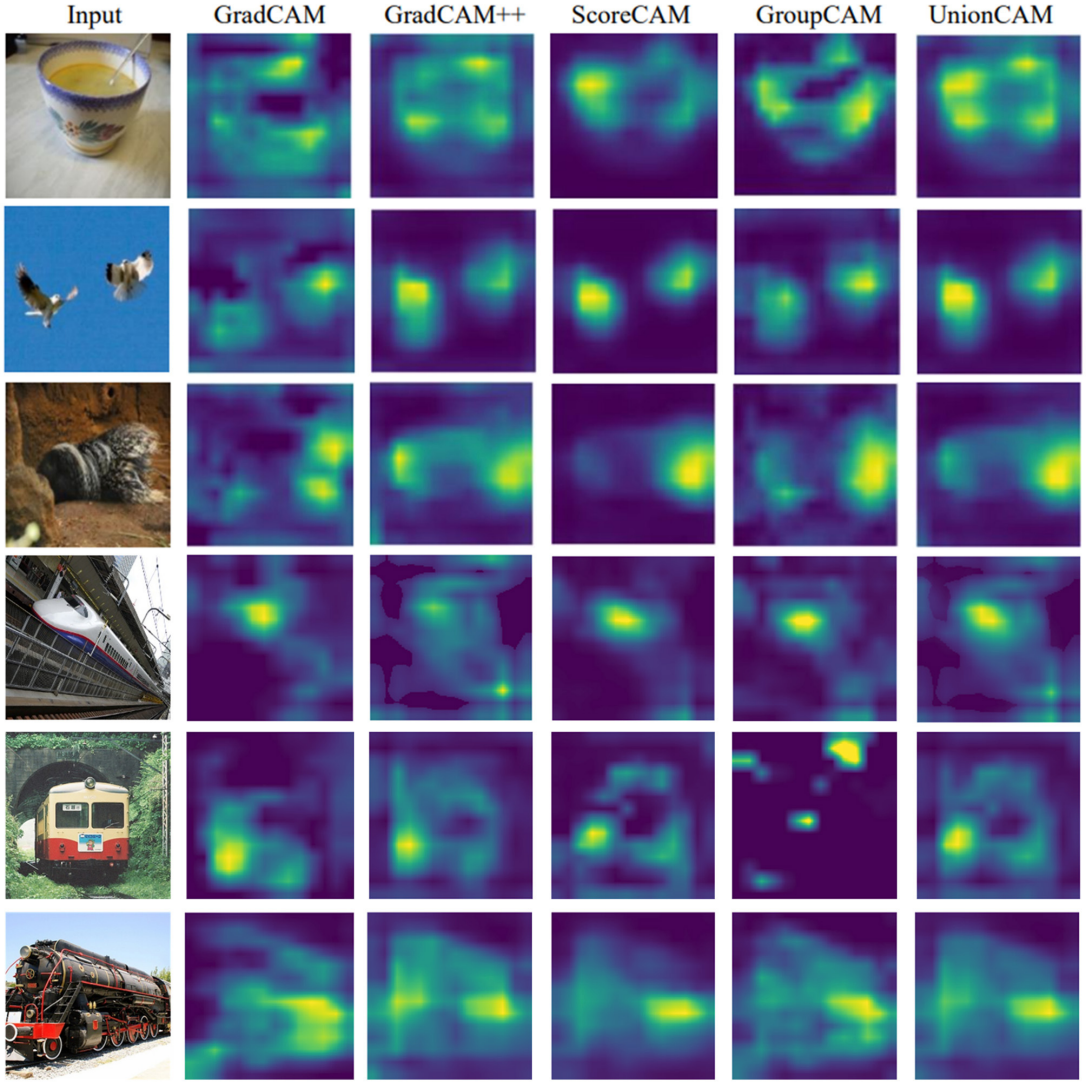
Visualization results of GradCAM, GradCAM++, ScoreCAM, GorupCAM, and UnionCAM.

We qualitatively compare the activation maps generated by our method with those from other state-of-the-art models. Our method produces activation maps that are relatively complete and exhibit less noise compared to those generated by GroupCAM and ScoreCAM. As shown in Figure 5, GradCAM sometimes focuses on irrelevant regions, leading to confusion in identifying key regions, such as the table area in the first row and the sky area in the second row. In contrast, GradCAM++ aims to concentrate more on relevant areas, but it may inadvertently neglect some meaningful regions, resulting in incomplete interpretations. For instance, in the fourth row, GradCAM++ has poor performance in capturing the meaningful area of the train. ScoreCAM occasionally has limited emphasis on target regions, as seen in the third row, potentially overlooking significant areas that contribute to the overall understanding of the model's decisions. GroupCAM sometimes fails to effectively focus on the target object, and its attention on large areas can dilute the focus on meaningful regions. In contrast, our method can often not only enhance the clarity of the activation maps but also ensure a more balanced focus on both relevant and meaningful regions. Our method effectively integrates useful information from different maps through a combination of soft and hard fusion techniques. This adaptive integration mechanism allows for a dynamic refinement of the activation maps, ensuring that the relevant and informative features are retained.

**Figure 6:**
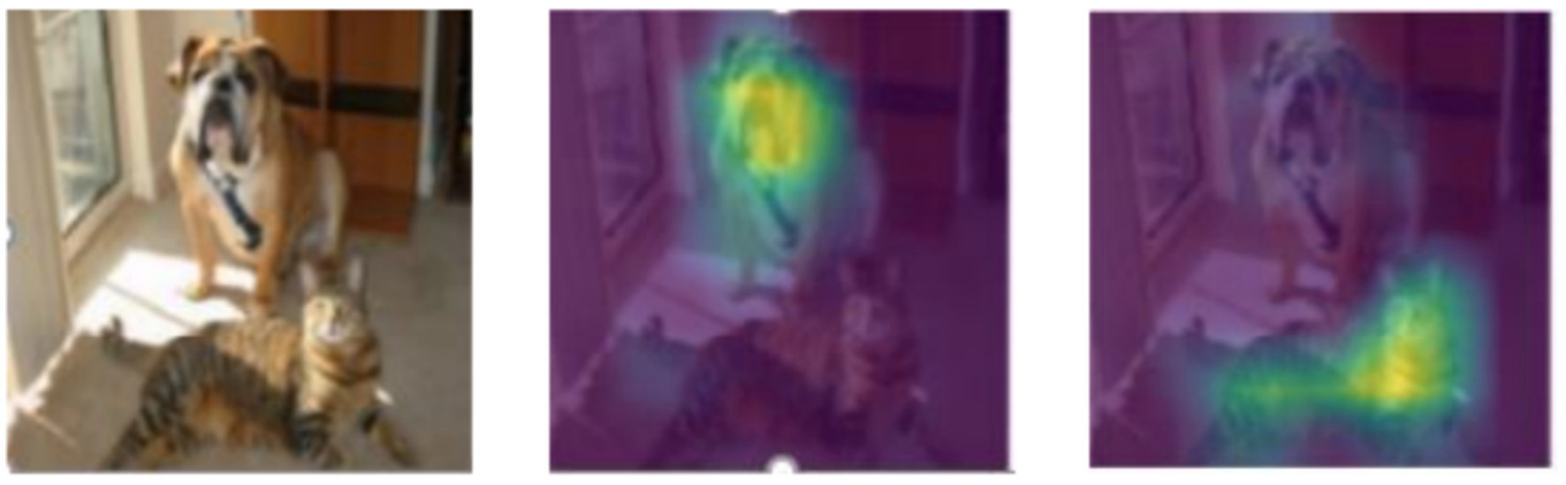
Category discrimination results. The middle graph is generated based on the input category of “bulldog,” and the graph on the right is generated based on the input category of “tabby cat.”

We further examine whether UnionCAM can distinguish between different classes. As shown in Figure 6, when VGG16 is used to classify the input as “bulldog” and “tabby cat,” UnionCAM provides distinct and accurate localization for each category, despite different confidence levels. As shown in Figure 6, VGG16 classifies the input as “bulldog” (47.08% confidence) and “tabby cat” (41% confidence). Although the confidence of the latter is lower than that of the former, UnionCAM can correctly provide the explanation positions corresponding to the two categories.

**Figure 7:**
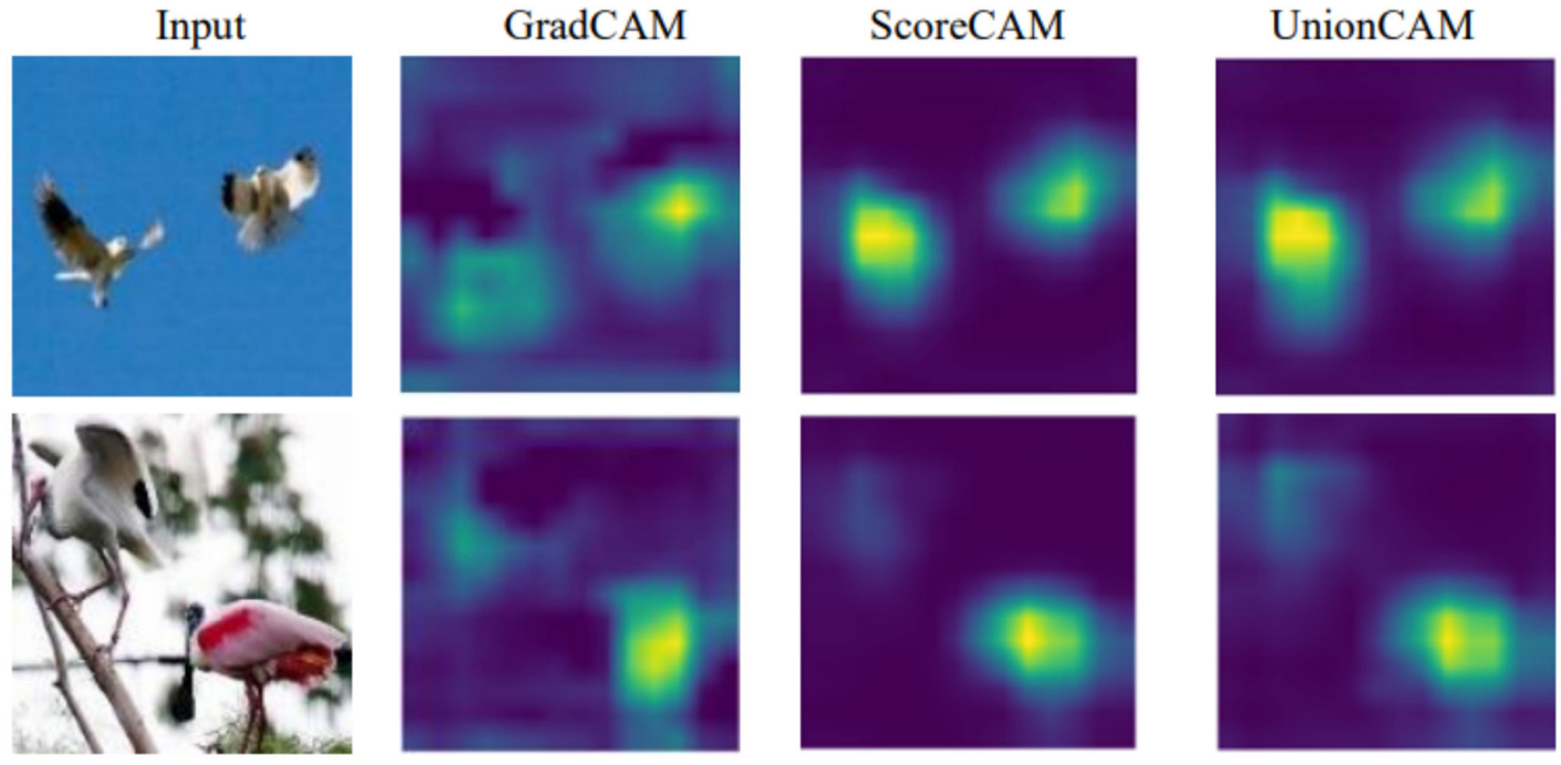
Multi-target detection results. From the results, GradCAM can usually locate only one object, while both ScoreCAM and UnionCAM can locate multiple objects, and UnionCAM is more interpretable.

UnionCAM not only accurately localizes single objects but also excels in identifying multiple objects within the same scene (two birds are located), outperforming previous methods. Figure 7 illustrates the superior multi-target detection capability of UnionCAM compared to GradCAM and ScoreCAM. However, the activation map generated by UnionCAM is more complete and focused compared to ScoreCAM.

### 4.4 Ablations

**Figure 8:**
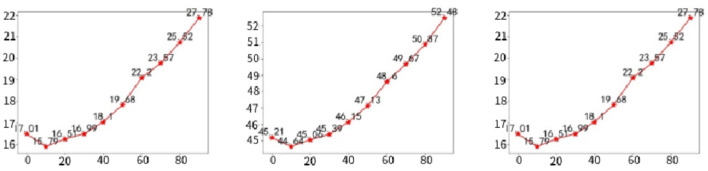
In the ImageNet (ILSVRC2012) validation set, the ablation experiments on the filter threshold *θ* are evaluated in terms of average drop (lower is better), average increase (higher is better), and over-all (lower is better) evaluation metrics performance.

We conduct ablation experiments on the ImageNet (ILSVRC2012) validation set to deeply investigate the effects of the denoising threshold θ and the activation map union weights β_*Denoising*_ and β_*Region*_ on the results. The experimental results are shown in Figure 8 and Table 3. The baseline at this stage is the network with only the Activation Map Selection (AMS) module added, and the Activation Map Union (AMU) module directly sums the two activation maps without any weighting.

From Figure 8, we can see that the threshold θ has a significant impact on the UnionCAM results. The *overall* score is calculated as *Average Drop*−*Average Increase*, so a lower value indicates better performance. When θ is relatively small, the *overall* score decreases. However, when θ>10, the *overall* score begins to increase sharply. To obtain better activation map quality, we set the default value of θ to 10.

We also experimented with adding weights β_*Denoising*_ and β_*Region*_ to combine the two activation maps in the AMU module, and compared the results with the baseline. The results show that both *Average Drop* and *Average Increase* have achieved better performance than the baseline after adding weights. The aggregated results are shown in Table 4.

**Table 4:** In the ImageNet (ILSVRC2012) validation set, comparisons are made in terms of deletion (lower is better), insertion (higher is better) scores, and overall (higher is better) evaluation metrics.

**Method**	**Average drop**	**Average increase**	**Overall**
Base (%)	45.21	28.20	17.01
Base + de-noising 10 (%)	44.64	28.85	15.79
Base + Weight (%)	43.71	28.75	14.96
Base + de-no + Weight (%)	**43.15**	**28.95**	**14.20**

The best results are marked in bold.

## 5 Conclusions

In this paper, we propose a novel visual interpretation method called UnionCAM for explaining the decision-making process of deep convolutional neural networks. UnionCAM addresses the limitations of existing methods by introducing a “denoising-union-selection” strategy to generate class activation maps (CAMs). The proposed method consists of three key modules: (1) an Activation Map Denoising (AMD) module to remove meaningless background noise from the gradient-based CAMs; (2) an Activation Map Union (AMU) module to combine the denoised CAMs with region-based CAMs using a learnable weight; and (3) an Activation Map Selection (AMS) module to adaptively select the most informative CAM for visual interpretation. We evaluate the proposed UnionCAM on two benchmark datasets, ILSVRC2012 and VOC2007, using four widely-used evaluation metrics: insertion, deletion, average drop, and average increase. The extensive experimental results demonstrate that UnionCAM outperforms the state-of-the-art methods by a significant margin. In particular, UnionCAM achieves a better balance between removing irrelevant background noise and preserving the complete object activation region, resulting in more accurate and human-interpretable visual explanations.

The proposed UnionCAM provides a novel perspective on interpreting the behavior of deep neural networks. By combining the strengths of both gradient-based and region-based methods, UnionCAM offers a more comprehensive and reliable approach to generate visual explanations. We believe that the insights gained from this work can facilitate the development of more transparent and trustworthy deep learning models, especially in critical domains such as healthcare and autonomous driving. While UnionCAM presents significant advantages, such as enhanced interpretability and improved activation map quality, it is important to also consider its limitations that may impact its effectiveness in various applications. The weighted fusion strategy, although effective, may struggle with complex scenes or overlapping objects, potentially leading to less accurate activation maps. This highlights the need for further refinement of the fusion mechanism to handle diverse visual challenges. In addition, The quality of region-based activation maps sometimes can impact the performance of the algorithm. Consequently, enhancing the quality of these maps is crucial for improving not only the interpretability but also the overall effectiveness of the algorithm.

In future work, we plan to extend UnionCAM to other types of neural networks, such as recurrent neural networks and graph neural networks, to provide a unified framework for interpretable deep learning. We will also explore the potential of integrating UnionCAM with other explanation techniques, such as feature visualization and concept activation vectors, to further enhance the interpretability of deep neural networks.

## Data Availability

The datasets presented in this study can be found in online repositories. The names of the repository/repositories and accession number(s) can be found in the article/supplementary material.
